# The Influence of Tumor Size on Oncologic Outcomes for Patients with Upper Tract Urothelial Carcinoma after Radical Nephroureterectomy

**DOI:** 10.1155/2016/4368943

**Published:** 2016-12-14

**Authors:** Xiaohong Su, Dong Fang, Xuesong Li, Gengyan Xiong, Lei Zhang, Han Hao, Yanqing Gong, Zheng Zhang, Liqun Zhou

**Affiliations:** Department of Urology, Peking University First Hospital, Institute of Urology, Peking University, National Urological Cancer Center, Beijing 100034, China

## Abstract

Previous studies have reached diverse conclusions about the influence of tumor size on the oncologic outcomes in patients with upper tract urothelial carcinoma (UTUC). In this study, we retrospectively analyzed the records of 687 patients and evaluated how tumor size affected the prognosis of patients with UTUC after surgery. Clinicopathologic characteristics and oncological outcomes were compared according to tumor size (≤3 cm versus >3 cm). During a median follow-up period of 65 months (range 3–144 months), 225 patients (32.8%) died from UTUC and 228 patients (33.2%) experienced intravesical recurrence (IVR). Patients with a larger tumor size tended to have a significantly higher percentage of being male (*p* = 0.011), tobacco consumption (*p* = 0.036), lack of preoperative ureteroscopy history (*p* = 0.003), renal pelvic location (*p* < 0.001), tumor necrosis (*p* = 0.003), advanced tumor stage (*p* < 0.001), higher tumor grade (*p* = 0.003), and lymph node metastasis (*p* = 0.018). Univariate analysis revealed that a tumor size >3 cm was significantly associated with worse cancer-specific survival (*p* = 0.002) and IVR (*p* = 0.011). However, the influence was not statistically significant after controlling for other factors in the multivariate analysis (hazard ratio [HR] 1.124, *p* = 0.414 and HR 1.196, *p* = 0.219). In conclusion, UTUC patients with a larger tumor present aggressive biological characteristics and tend to have a worse prognosis.

## 1. Introduction

Upper tract urothelial carcinomas (UTUCs) are rare and account for only 5% to 10% of urothelial carcinomas [1]. UTUC patients generally have more advanced disease at the time of diagnosis than bladder cancer [2, 3], and a poor prognosis is usually identified in advanced local disease, with a 5-year cancer-specific survival (CSS) less than 50% for pT2/pT3 and <10% for pT4 [4, 5]. Another important feature of UTUC is the high risk of intravesical recurrence (IVR) after radical surgery, with the reported recurrence rate varying considerably from 22% to 47% [6–8]. Thus far, no high-volume perspective study has confirmed the potential prognostic factors of UTUC because of the small prevalence of this malignancy. According to previous studies, several prognostic factors have been investigated to help urologists identify patients who are at a high risk of worse oncologic outcomes and then schedule a stringent follow-up regimen. Older age, advanced tumor stage and grade, pelvic location, lymphovascular invasion (LVI), and positive lymph nodes are documented as risk factors in patients with UTUC [9].

Tumor size has been recognized as a risk factor for poor oncologic outcomes in urothelial carcinoma of the bladder, while the prognostic influence of the tumor size in UTUC has not been fully addressed. A few studies [10–13] about the impact of tumor size on the oncologic outcome in UTUC patients have recently been performed. However, these studies have reached diverse conclusions due to their retrospective and small-volume nature.

The aim of this high-volume study is to assess the association between tumor size and biological characteristics and validate the impact of tumor size on the oncological outcomes of UTUC patients treated with radical nephroureterectomy (RNU).

## 2. Patients and Methods

### 2.1. Patient Selection

Between Aug 1, 1999, and Dec 31, 2011, a total of 820 patients underwent operations for clinically localized UTUC. Seventy-six patients with concomitant or previous bladder tumors, 22 patients with metastatic disease, and 35 patients with incomplete follow-up data were excluded from this study. After obtaining institutional review board approval, the clinical and pathological data for the remaining 687 patients were retrospectively analyzed.

### 2.2. Treatment and Evaluation

In this cohort, all patients received RNU with the excision of the ipsilateral bladder cuff. The definitions of routine lymph node dissection, tumor stage, tumor grade, and tumor size were described in our previous study [14]. In this study, none of the patients received neoadjuvant chemotherapy. For those patients in whom the final pathological analysis confirmed advanced tumor stage, positive lymph node, or retroperitoneal recurrence, adjuvant radiotherapy or chemotherapy was suggested.

### 2.3. Follow-Up Regimen

During the follow-up period, cystoscopy, laboratory tests, chest X-ray, and urological ultrasound were performed every 3 months during the first 2 years, which extended to a semiannual period for the next 2 years and once a year thereafter. Abdominal and pelvic computed tomography or magnetic resonance imaging scans were carried out annually. Follow-ups were censored until their last visit or death.

### 2.4. Statistical Analysis

SPSS version 20.0 (IBM Corp, Armonk, NY) was used for all statistical analyses and a *p* < 0.05 was considered significant. Chi-squared and Mann–Whitney *U* tests were used for the categorical variables. Kaplan-Meier method with the log-rank test was used to assess recurrence-free survival and CSS. Multivariate analyses were conducted using Cox's proportional hazard model.

## 3. Results

### 3.1. Clinical and Pathological Features

The clinicopathologic demographics of the patients are listed in Table 1 and are stratified according to the tumor size. Three hundred and eighty-five patients (56%) had a tumor ≤3 cm and 302 patients (44%) had a tumor >3 cm. There were no significant differences in terms of age, surgical approach, hydronephrosis, tumor side, concomitant CIS, tumor architecture, tumor focality, and mixed histologic variant according to tumor size. However, patients with tumors >3 cm tended to be male (50% versus 40.3%, *p* = 0.011) and had a significantly higher percentage of tobacco consumption (21.5% versus 15.3%, *p* = 0.036), lack of preoperative ureteroscopy history (8.3% versus 15.8%, *p* = 0.003), renal pelvic location (63.6% versus 48.85, *p* < 0.001), and tumor necrosis (15.6% versus 8.3%, *p* = 0.003). Significantly higher rates of advanced pathologic *T* stage (*p* < 0.001), tumor grade (*p* = 0.003), and positive lymph node (*p* = 0.018) were present in the cohort of patients with a tumor >3 cm.

### 3.2. Oncologic Outcomes

**Figure 1:**
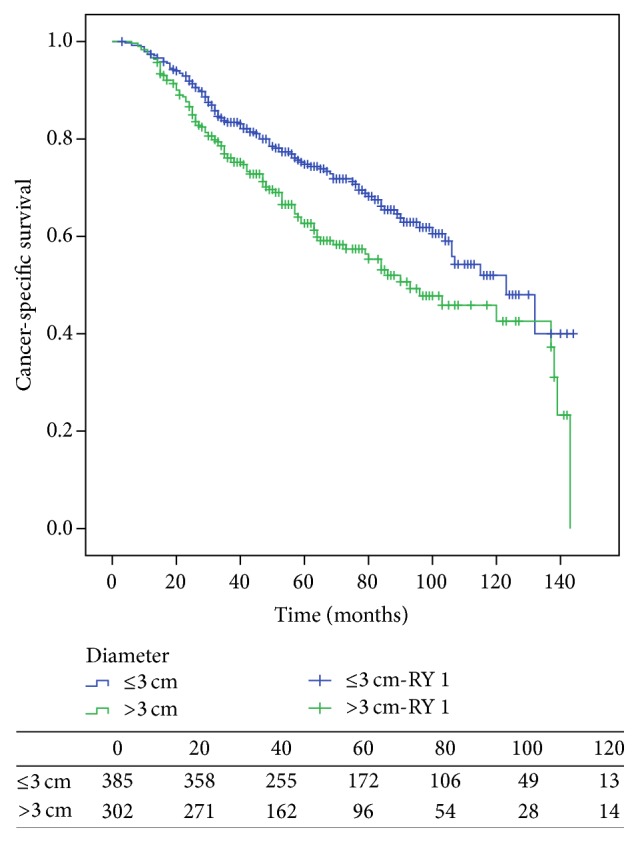
Estimated Kaplan-Meier cancer-specific survival curve stratified by tumor size (*p* = 0.002).

The median follow-up time was 65 months (range 3–144 months). In this cohort, 225 patients (32.8%) died from UTUC, including 112 patients with a tumor ≤3 cm and 113 patients with a tumor >3 cm, and bladder recurrence was found in 74 (10.8%) of this cohort of patients. The five-year CSS was 74.8% for patients with a tumor ≤3 cm and 62.6% for patients with a tumor >3 cm (*p* = 0.002; Figure 1). The estimated median CSS time was 123 months for patients with smaller tumors and 93 months for patients with larger tumors. Pathology confirmed 228 patients (33.2%) with IVR within a median time of 17 months (range 2–102 months). One hundred and forty-six (64.0%) IVR cases occurred within 2 years after RNU.

In the univariate analysis (Table 2), tumor size (*p* = 0.002) and several other variables were significant risk factors for a worse CSS (all *p* < 0.05). In the multivariate analysis, however, after adjusting for other clinicopathologic characteristics, tumor size was not a significant independent predictor of worse CSS (*p* = 0.414, HR 1.124). Rerunning the database with 4 cm or 5 cm as the cut-off value did not change the results. Older age (*p* = 0.007, HR 1.455), male gender (*p* = 0.001, HR 1.600), presence of hydronephrosis (*p* < 0.001, HR 1.675), advanced tumor stage (*p* < 0.001, HR 2.266), and positive lymph nodes (*p* = 0.006, HR 1.815) were independent risk factors for cancer-related mortality (Table 2).

**Figure 2:**
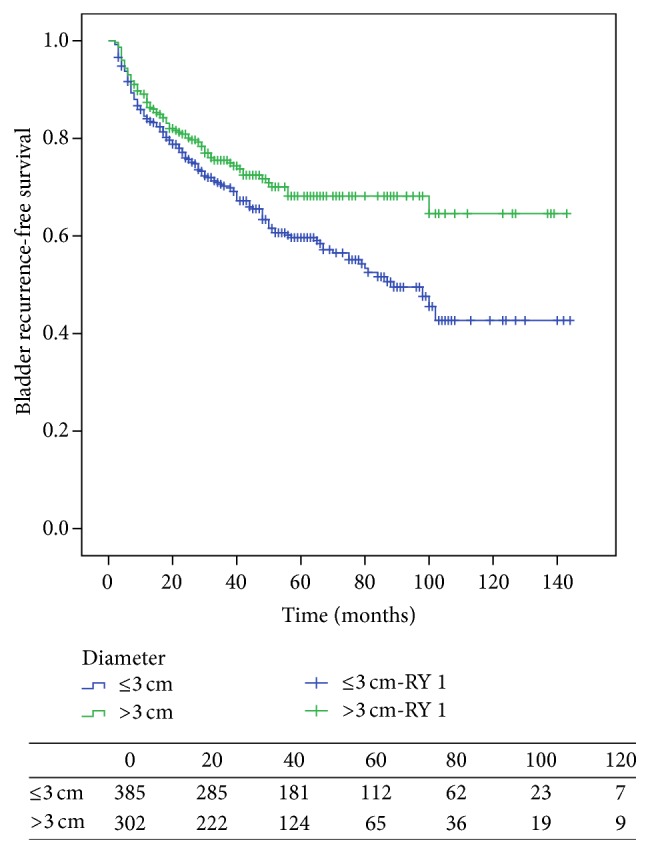
Estimated Kaplan-Meier bladder recurrence-free survival curve stratified by tumor size (*p* = 0.011).

The tumor size was inversely related to the risk of IVR. Using Kaplan-Meier method, the bladder recurrence-free rate of a larger tumor size tended to be greater than that of a smaller tumor size (*p* = 0.011, by the log-rank test; Figure 2). Using univariate analysis (Table 3), smaller tumor size, preoperative ureteroscopic history, lower pathologic T stage, lower tumor grade, cN0 or pN0 status, tumor multifocality, and ureteral tumors were correlated with IVR. Using multivariate analysis, preoperative ureteroscopic history (*p* = 0.002, HR 1.656), lower tumor grade (*p* = 0.032, HR 1.391), cN0 or pN0 status (*p* = 0.020, HR 3.279), and tumor multifocality (*p* = 0.001, HR 1.601) remained as prognostic factors for recurrence (Table 2).

If we excluded 151 patients who died before developing bladder recurrence during the follow-up period, smaller tumor size was not a risk factor for increased bladder recurrence by univariate analysis (*p* = 0.139); only preoperative ureteroscopic history and tumor multifocality remained independent prognostic factors for bladder recurrence (*p* = 0.011 and *p* = 0.001, resp.).

## 4. Discussion

In the present study, we found that larger tumors were associated with higher rates of male patients, tobacco consumption, no preoperative ureteroscopic history, pelvic tumor location, tumor necrosis, advanced tumor stage, higher tumor grade, and lymph node metastasis. Larger tumors may present in men as they often delay presentation with hematuria. Larger tumors are less likely to undergo ureteroscopy as CT imaging is more convincing in identifying urothelial malignancy and therefore ureteroscopy may not be required. Larger tumors are more likely to present in the renal pelvis, as they would be symptomatic with pain and obstruction in the ureter before they reach this size.

The Kaplan-Meier curve showed that patients with tumors >3 cm had worse CSS, and univariate analysis also confirmed large tumor size as a prognostic risk factor. A recent study confirmed that the tumor size was an independent predictor for worse CSS and overall survival [15]. However, in our research, after adjusting for clinicopathologic variables, the impact of tumor size on CSS was not statistically significant by multivariate analysis, in accordance with Milenkovic-Petronic's study [10].

In this cohort, patients with a tumor size >3 cm had a higher rate of having biological aggressive characteristics, including advanced tumor stage, higher tumor grade, lymph node metastasis, and tumor necrosis. Zigeuner et al. confirmed that extensive tumor necrosis was independently associated with disease recurrence and survival (*p* = 0.037 and *p* = 0.046, resp.) [16]. Simone et al. [17] reported that all the metastasis and cancer-related deaths occurred in patients with extensive tumor necrosis (≥10% tumor area) and a tumor diameter ≥3 cm.

The rate of IVR was 33.2%, in accordance with previous studies [6–8]. The median interval to IVR was 17 months and >60% of recurrences occurred within two years after surgery. Many risk factors contributing to IVR had been examined in previous studies and were considered in making optimal clinical decisions. However, the exact cut-off value and influence of tumor size on IVR remain controversial. In this study, we recognized smaller tumor size (≤3 cm) as an adverse predictor of subsequent IVR in univariate analysis which was not in accordance with several previous studies [10–12, 15, 18]. Our previous study [18] and that of Milenkovic-Petronic et al. [10] showed that tumor size was not able to predict IVR up on univariate and multivariate analysis when tumor sizes of 5 cm and 3 cm were set as the cut-off value, respectively. Pieras et al. [12] and Espiritu et al. [11] confirmed that patients with a tumor size >4 cm or ≥3 cm had a higher rate of IVR. In a recent study with a larger-volume Chinese population, Yan et al. [15] reported the influence of tumor size on prognostic outcomes for UTUC patients. They found that patients with a tumor size >3 cm showed a poor disease recurrence-free survival. As a larger tumor has more surface area, the intraluminal seeding theory might play a more important role in the development of IVR. However, Matsui et al. [13] reported that smaller tumors (<2 cm versus 2–5 cm versus >5 cm) increased the risk of IVR.

The different finding requires convincing explanation. One reason for this inverse impact on oncologic outcome may be that larger tumor size is correlated with a high cancer-specific mortality and patients may die before the development of a bladder tumor. Patients with smaller tumor size may have a longer lifespan so that more IVR can be found. After excluding 151 patients who died without bladder recurrence, the impact of tumor size was not statistically significant, with *p* = 0.139. In our research, the impact of tumor size on IVR was not supported by multivariate analysis. Although the impact of tumor size on IVR is controversial, we still suggest that a stringent surveillance plan for postoperative IVR is needed for patients with larger tumors. A more personalized risk-based follow-up scheme and potential adjuvant treatment might be possible after consideration of tumor size. And we would be closer to precise patients' risk-stratification with more understanding of these risk factors.

RNU with the excision of an ipsilateral bladder cuff is the gold-standard treatment for UTUC [9]. The use of endoscopic management for the treatment of UTUC with low stage and grade increased in recent years due to advances in technology [19]. However, it is difficult to determine the correct tumor stage and grade of UTUC because of the small and ablated sampling specimen via preoperative ureteroscopy. In fact, UTUCs are more likely to be locally invasive and exhibit distant metastasis, probably because of the thinner muscle layer structure [20] and abundant lymphatic and blood channels [21] of the upper urinary tract. Because the association between tumor size and biologically aggressive features has been confirmed in many studies and advances in preoperative radiographic imaging make it possible to accurately measure the tumor size preoperatively, we must pay more attention to tumor size and a prospective study should be performed to determine a more satisfying cut-off value for tumor size.

Our study has some limitations. First, this is a retrospective and single-center study. Second, none of the patients, especially those with advanced tumor stage and grade, received neoadjuvant chemotherapy. Finally, the lack of information on postoperative chemotherapy and radiotherapy would influence patient survival. Nonetheless the current study is still one of the largest single-center cohorts of UTUC patients, and the detailed analysis with regard to each clinical and pathological feature based on this large sample could provide more convincing information to clinicians.

## 5. Conclusions 

Patients with a tumor size >3 cm showed a higher rate of aggressive biological characteristics and tended to have worse CSS. A prospective and large-scale study is urgently needed to define the impact of tumor size and find a more satisfying cut-off value for tumor size.

## Figures and Tables

**Table 1:** Clinical and pathological characteristics of 687 patients with UTUC stratified by tumor size.

		Tumor size			
		≤3 cm	>3 cm	Chi-square or *Z*	*p* value^*∗*^
	All	385 (%)	302 (%)		
Age	≤70	221 (57.4)	188 (62.3)	1.652	0.199
	>70	164 (42.6)	114 (37.7)		
Median (range)		69 (20–90)	68 (29–86)	−1.424	0.155
Gender	Male	155 (40.3)	151 (50.0)	6.500	0.011^*∗*^
	Female	230 (59.7)	151 (50.0)		
Tobacco consumption	Yes	59 (15.3)	65 (21.5)	4.396	0.036^*∗*^
	No	326 (84.7)	237 (78.5)		
Surgical approach	Open	127 (33.0)	93 (30.8)	0.374	0.541
	Laparoscopic	258 (67.0)	209 (69.2)		
Preoperative ureteroscopy	Yes	61 (15.8)	25 (8.3)	8.847	0.003^*∗*^
	No	324 (84.2)	277 (91.7)		
Hydronephrosis	Presence	208 (54.0)	126 (41.7)	1.241	0.265
	Absence	177 (46.0)	176 (58.3)		
T stage	PTis-a-1	165 (42.9)	64 (21.2)	59.636	<0.001^*∗*^
	PT2	140 (36.4)	102 (33.8)		
	PT3	77 (20.0)	120 (39.7)		
	PT4	3 (0.8)	16 (5.3)		
Grade	G1	16 (4.2)	5 (1.7)	11.654	0.003^*∗*^
	G2	222 (57.7)	146 (48.3)		
	G3	147 (38.2)	151 (50.0)		
Lymph node status	cN0 or pN0	363 (94.3)	270 (89.4)	5.569	0.018^*∗*^
	N+	22 (5.7)	32 (10.6)		
Main tumor location	Ureter	197 (51.2)	110 (36.4)	14.886	<0.001^*∗*^
	Pelvis	188 (48.8)	192 (63.6)		
Side of tumor location	Right	189 (49.1)	150 (49.7)	0.023	0.880
	Left	196 (50.9)	152 (50.3)		
Concomitant CIS	Presence	10 (2.6)	10 (3.3)	0.305	0.581
	Absence	375 (97.4)	292 (96.7)		
Tumor architecture	Papillary	302 (78.4)	228 (75.5)	0.832	0.362
	Sessile	83 (21.6)	74 (24.5)		
Multifocality	Yes	97 (25.2)	67 (22.2)	0.843	0.358
	No	288 (74.8)	235 (77.8)		
Tumor necrosis	Presence	32 (8.3)	47 (15.6)	8.744	0.003^*∗*^
	Absence	353 (91.7)	255 (84.4)		
Squamous and/or glandular differentiation	Presence	38 (9.9)	43 (14.2)	3.105	0.078
	Absence	347 (90.1)	259 (85.8)		

eGFR: estimated glomerular filtration rate; CIS: carcinoma in situ.

^*∗*^Statistically significant.

**Table 2:** Univariate and multivariate analyses of predictive factors for worse cancer-specific survival.

	Univariate analysis			Multivariate analysis		
	HR	95% CI	*p*	HR	95% CI	*p*
Age (>70 versus ≤70)	1.417	1.088–1.846	0.010^*∗*^	1.455	1.107–1.912	0.007^*∗*^
Gender (male versus female)	1.681	1.291–2.188	<0.001^*∗*^	1.600	1.222–2.096	0.001^*∗*^
Tobacco consumption (yes versus no)	0.982	0.697–1.382	0.915			
Surgical approach (open versus laparoscopic)	1.035	0.764–1.404	0.823			
Preoperative ureteroscopy (yes versus no)	0.517	0.323–0.828	0.006^*∗*^	0.630	0.391–1.014	0.057
Hydronephrosis (presence versus absence)	1.415	1.081–1.854	0.012^*∗*^	1.675	1.262–2.224	<0.001^*∗*^
T stage (pT3-4 versus pTis-a-1-2)	2.569	1.973–3.345	<0.001^*∗*^	2.266	1.643–3.126	<0.001^*∗*^
Tumor grade (G3 versus G1-2)	1.560	1.201–2.028	0.001^*∗*^	0.824	0.583–1.166	0.275
Lymph node status (Nx versus cN0 or pN0)	2.627	1.785–3.867	<0.001^*∗*^	1.815	1.191–2.767	0.006^*∗*^
Tumor size (>3 cm versus ≤3 cm)	1.507	1.159–1.959	0.002^*∗*^	1.124	0.849–1.489	0.414
Main tumor location (ureter versus pelvis)	1.139	0.876–1.482	0.331			
Concomitant CIS (presence versus absence)	1.673	0.912–3.069	0.096			
Tumor architecture (sessile versus papillary)	1.942	1.458–2.587	<0.001^*∗*^	1.197	0.823–1.742	0.347
Focality (multiple versus single)	1.149	0.858–1.538	0.352			
Tumor necrosis (presence versus absence)	1.913	1.336–2.739	<0.001^*∗*^	1.357	0.913–2.016	0.131
Squamous and/or glandular differentiation (presence versus absence)	1.957	1.346–2.846	<0.001^*∗*^	1.403	0.927–1.823	2.124

eGFR: estimated glomerular filtration rate; CIS: carcinoma in situ.

^*∗*^Statistically significant.

**Table 3:** Univariate and multivariate analyses of predictive factors for bladder recurrence survival.

	Univariate analysis			Multivariate analysis		
	HR	95% CI	*p*	HR	95% CI	*p*
Age (≤70 versus >70)	0.899	0.687–1.176	0.439			
Gender (male versus female)	1.159	0.893–1.504	0.268			
Tobacco consumption (yes versus no)	1.030	0.737–1.439	0.864			
Surgical approach (open versus laparoscopic)	1.042	0.783–1.387	0.776			
Preoperative ureteroscopy (yes versus no)	2.085	1.527–2.847	<0.001^*∗*^	1.656	1.196–2.291	0.002^*∗*^
Hydronephrosis (presence versus absence)	1.253	0.962–1.632	0.095			
T stage (pTis-a-1-2 versus pT3-4)	1.462	1.071–1.996	0.017^*∗*^	1.034	0.730–1.464	0.850
Tumor grade (G1-2 versus G3)	1.587	1.201–2.092	0.001^*∗*^	1.391	1.028–1.880	0.032^*∗*^
Lymph node status (cN0 or pN0 versus Nx)	4.525	1.684–12.195	0.003^*∗*^	3.279	1.202–8.929	0.020^*∗*^
Tumor size (≤3 cm versus >3 cm)	1.422	1.082–1.869	0.012^*∗*^	1.196	0.898–1.595	0.219
Main tumor location (ureter versus pelvis)	1.396	1.076–1.811	0.012^*∗*^	1.253	0.955–1.646	0.104
Concomitant CIS (presence versus absence)	1.155	0.571–2.340	0.688			
Tumor architecture (papillary versus sessile)	0.739	0.521–1.047	0.089			
Focality (multiple versus single)	1.813	1.381–2.381	<0.001^*∗*^	1.601	1.213–2.114	0.001^*∗*^
Tumor necrosis (presence versus absence)	0.826	0.527–1.294	0.404			
Squamous and or glandular differentiation (presence versus absence)	0.682	0.416–1.119	0.130			

eGFR: estimated glomerular filtration rate; CIS: carcinoma in situ.

^*∗*^Statistically significant.
